# Research on the resource and governance effects of state-owned equity participation: Based on the analysis of the green transformation process and path of private enterprises

**DOI:** 10.1371/journal.pone.0337838

**Published:** 2025-12-05

**Authors:** Haizhi Ren, Yani Wen

**Affiliations:** School of Business Administration, Liaoning Technical University, Huludao City, Liaoning Province, China; Sichuan University, CHINA

## Abstract

Against the backdrop of environmental challenges and resource constraints, deepening mixed ownership reform and promoting green development of enterprises have become a significant trend, and the resource and governance effects arising from the state-owned (SOE) equity participation in private enterprises (PEs) have become a prominent research topic. The article empirically examines the influence of state-owned equity involvement on the green transformation of private firms using a two-way fixed effects model, utilizing a sample of Shanghai and Shenzhen A-share listed private enterprises from 2013 to 2022. The results indicate that SOE equity participation considerably speeds up the green transformation process of PEs, and the conclusion remains consistent following several robustness tests. Through the two channels of financing efficiency and environmental information disclosure, SOE equity participation encourages the green transformation of PEs; management compensation incentives have a positive moderating effect on SOE equity participation and the green transformation of PEs. According to heterogeneity analyses, SOE ownership has a stronger motivating effect on PEs’ green transformation for large-scale, high-tech, and eastern area firms. The findings enrich the inquiry into the economic effects of reverse mixed reform and establish practical methods and theoretical foundations for private enterprises to promote green transformation.

## 1. Introduction

Green transformation, or the transition to a resource-conservative and ecologically friendly development paradigm, has become a crucial means for private enterprises to achieve sustainable development. In the context of globalization and industrialization, the swift rise of China’s private enterprises has helped the country’s GDP surpass 60%, making it a major driver of economic expansion. However, while pursuing economic interests, private enterprises have neglected issues such as resource consumption and environmental pollution, and the traditional development model of high pollution and energy consumption has become unsustainable. For instance, in 2023, China’s total energy consumption will reach 5.72 billion tonnes of standard coal, up 5.7 percent year on year; the average annual concentration of PM2.5 will be 30 micrograms per cubic meter, up 3.4 percent year on year. “Advancing the greening and low-carbon transition of both the economy and society is an essential element in the pursuit of high-quality development,” the Twentieth Report boldly asserts. Given their substantial contribution to China’s carbon dioxide emissions, the green transformation of PEs is integral to achieving the nation’s dual-carbon goals. To speed up China’s high-quality economic development and successfully implement the green development plan, discerning the intrinsic mechanisms of green transformation within private enterprises is a prerequisite for actualizing these objectives.

Academics are directing their research toward the green transition, which encompasses a multitude of levels and dimensions. Initially, the emphasis was placed on the strategy, modalities, and trajectory of green transformation. Acemoglu et al. (2012) [1] advocated for the implementation of a combined policy approach involving pollution charges and innovation subsidies to foster green technology innovation and energy efficiency. Zhang et al. (2015) [2] proposed to effectively promote industrial green transformation by strengthening top-level design, enhancing organizational implementation, and improving support policies. As research progresses, scholars have started to concentrate on studying the drivers of green transformation at the enterprise level, mainly examining the influence of the external institutional environment and internal corporate governance, specifically in terms of environmental regulation, environmental dynamics, tax reduction incentives, media attention, organizational resource redundancy, executives environmental experience, and board governance with the green transformation of enterprises. Research findings indicate that, while there has been a modest improvement in the level of green transformation among private enterprises, they still face two internal problems: insufficient transformation motivation and transformation ability. How to stimulate the intrinsic green transformation capabilities of private enterprises? Few scholars have focused on the mixed ownership reform, which is crucial for enhancing market vitality and promoting the green transformation of PEs.

**Fig 1:**
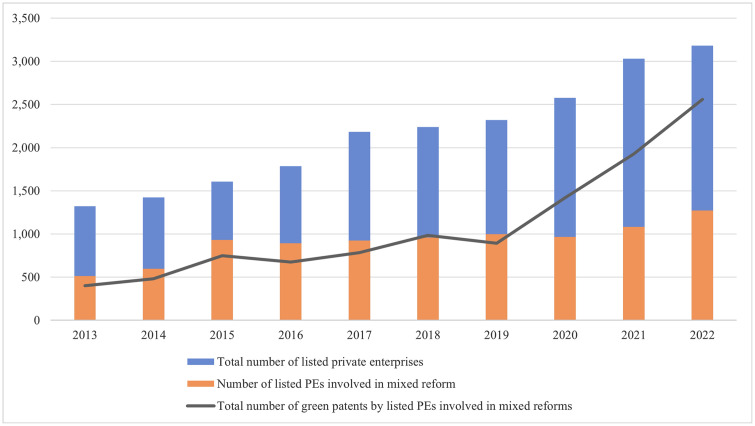
Trends of mixed reform participation and green patent applications among A-share listed PEs in China from 2013 to 2022.

As mixed ownership reform advances into a comprehensive deepening phase, bidirectional integration emerges as a crucial developmental trajectory. It not only covers the path of “Forward mixing reform”, which encourages non-state capital to participate in state-owned enterprises (SOEs) but also heralds a new chapter of “Reverse mixed reform”, which guides SOE capital to flexibly adopt a variety of ways to participate in PEs. Since 2013, the Chinese government has promulgated multiple policies to incentivize PEs’ participation in mixed ownership reform. Driven by these policies, an increasing number of private firms enhance their operational milieu through state capital infusion, thereby fostering their robust and sustainable growth (Pilla et al., 2025) [3]. Fig 1 shows the trend of private enterprise participation in mixed reform and green patent applications from 2013 to 2022. So, can the participation of state shareholders promote the green transformation of PEs? If so, what is the mechanism? The theoretical community has not yet concluded. The clear determination and reasonable solution of this series of issues can furnish a crucial theoretical basis and policy suggestions for deepening the mixed ownership reform and promoting the green transformation of PEs in China. Based on this, the article selects Shanghai and Shenzhen A-share listed private enterprises from 2013 to 2022, empirically examines the effect of SOE equity participation on the green transformation of PEs, and analyses the mediating role of financing efficiency and environmental information disclosure, the moderating effect of management compensation incentives, and the heterogeneous impacts of SOE equity participation on the green transformation of PEs across different scenarios.

When contrasted with existing research, this article may make the following contributions: (1) From the perspective of equity structure, it provides a novel analytical framework for the study of private enterprises’ green transformation, and effectively broadens the breadth and depth of research on the beneficial effects that PEs provide for the environment and society. (2) It delves into the economic implications of mixed ownership reform, emphasizes the perspective of PEs’ participation in mixed reform, and analyzes in depth the advantageous contribution of state capital to the green transformation of PEs, which further enriches the research in the field related to mixed reform. (3) In the context of the current national efforts to promote the acceleration of environmental civilization and high-quality synergistic development of the economy, the findings provide valuable guidance for the green transformation of PEs, thereby facilitating their pursuit of sustainable development.

## 2. Literature review

### 2.1 Research on the causes and effects of “Reverse mixed reform”

State-owned capital has increasingly become a “stabilizer” for the robust advancement of economic and social and a “ballast” for elevating public welfare. As a synthesis of political and economic governance systems, inquiries into mixed ownership reform at the corporate level have garnered significant attention within academic spheres. However, the prevailing literature mainly concentrates on “Forward mixing reform”, and pays relatively little attention to “Reverse mixed reform”. The majority of analyses initiate from political affiliations, ignoring the intrinsic governance perspective of private enterprises.

#### (1) Study on the motivation for “Reverse mixed reform”.

At present, the motivation for “Reverse mixed reform” can be discussed from governmental and private enterprise perspectives. From the government level, firstly, the government aims to exert strategic control over pivotal technological sectors and critical domains, ensuring the rapid and robust growth of the national economy, and achieving predetermined goals by utilizing the political affiliation advantage of the state-owned capital. Secondly, due to the relatively weak risk-bearing and crisis-response capacity of private enterprises, the government will assist private enterprises to cope with major crises, safeguard the smooth operation of the economy, and compensate for their inherent shortcomings through state-owned equity participation. From the private enterprise level: firstly, private enterprises confront myriad challenges in the process of development, including credit bias and financial limitations. The introduction of SOE capital can alleviate these financing difficulties and provide substantial support in terms of credit resources, tax incentives, government subsidies, etc., which in turn enhances the innovative capabilities of enterprises (Xu et al., 2024) [4]. Secondly, because private enterprises face a more complex external milieu, the local government usually interferes with them, so private enterprises seek property rights protection through the participation of SOE equity (Huang et al., 2025) [5].

#### (2) Study on the effects of “Reverse mixed reform”.

The existing literature focuses on both resource and governance effects.

Resource effect: SOE capital forms an institutional association with private firms, which in turn helps private firms obtain more resource support. Li et al. (2025) [6] propose that such participation enables enterprises to secure greater government subsidies, thereby enhancing their investment in R&D and innovation; Abdeljawad et al. (2024) [7] argue that SOE equity engagement can mitigate the financing constraints encountered by PEs and reduce the principal-agent cost; Li et al. (2023) [8] demonstrate that state-owned equity involvement has broadened the relationship network of private enterprises, including the bank-enterprise and the government-enterprise network; Yang et al. (2023) [9] assert that SOE equity upgrades the cash-holding behavior of private enterprises while enhancing their value. Bai et al. (2021) [10] argued that “reverse mixed reform” significantly reduces the cost of financing and expands the scale of financing by enhancing corporate reputation, optimizing information quality, and improving corporate governance.

Governance effects: SOE capital investment in PEs helps build a diversified shareholding structure, facilitates equity checks and balances, and alleviates the agency problem of “majority-minority shareholder” in private enterprises, thereby enhancing corporate governance standards. Yu and Qi (2022) [11] posit that the state-owned shareholder involvement strengthens the supervision of the operational activities of private enterprises and increases the likelihood of being audited, thus reducing the tendency of private enterprises to violate the law; Ren et al. (2024) [12] contend that the state-owned capital weakens the financing incentives for financialization, thereby restraining the real enterprises from becoming virtual; Chen et al. (2023) [13] propose that “reverse mixed reform” reduces the tax evasion degree among PEs by alleviating financing constraints, reducing agency costs, and enhancing social responsibility.

The preceding research findings indicate that state capital involvement positively affects the sustained growth of private enterprises, particularly in terms of resource acquisition and corporate governance. Can state-owned equity participation inject new vitality into the green transformation of private enterprises and promote their healthy and sustainable development? The theoretical community is still inconclusive.

### 2.2 Research on the factors influencing green transformation of PEs

China’s swift economic advancement has resulted in significant ecological and environmental challenges, notably greenhouse gas emissions and air pollution. Currently, the concept of green transformation has gained widespread acceptance as a collective strategy to tackle the issues of climate change, environmental pollution, and ecological degradation. Furthermore, it is viewed as a vital opportunity to advance the future trajectory of the economy and society (Farza et al., 2022) [14]. Concerning the determinants affecting the green transformation of PEs, the existing literature has primarily focused on two dimensions: the external institutional environment and internal corporate governance.

#### (1) The influence of the external institutional environment.

Existing studies, predominantly grounded in institutional and stakeholder theories, have dissected the impact of various factors, including the government, market and industry environments, and the public. Sun et al. (2024) [15] find that government guidance funds have significantly accelerated the green transformation of enterprises; Aftab et al. (2023) [16] contend that environmental regulation promotes corporate green transformation by augmenting the penalties for environmental violations and reducing the operational efficiency of corporate violations; Zhong and Peng (2022) [17] suggest that the adoption of voluntary environmental regulation positively modulates green technological innovation by mitigating public endorsement and fostering collaboration with external entities; Tian et al. (2022) [18] proposed that green credit policy (GCP) can effectively redirect financial resources from high-energy-consuming and high-polluting sectors to technology-driven emerging industries, thereby bolstering corporate green transformation; Chen et al. (2022) [19] argued that media scrutiny can diminish information asymmetry among stakeholders and positively affect corporate green transformation, and adverse environmental reports can compel enterprises to undertake green innovation.

#### (2) The impact of internal corporate governance mechanisms.

Existing studies, anchored in resource dependence theory and upper echelons theory, have mainly explored the impact of internal firm factors, including resource capacity, executive characteristics, and production technology. Chen and Xiao (2025) [20] proposed that the rational use of organizational redundancy resources can enhance corporate green innovation initiatives and positively influence their green transformation; Feng et al. (2024) [21] argued that transparency in environmental disclosure helps to create a positive image of the firm and attracts sustainability-focused investors, which in turn injects funds into green technology R&D; Song et al. (2020) [22] found that effective board governance can facilitate objective and impartial strategic decision-making that supports a company’s long-term growth; Ding et al. (2023) [23] suggest that the allocation of executive environmental attention (EEA) can promote the ecological advancement of industrial configurations, thereby actualizing the corporate transition towards sustainable development paradigms; Zhang et al. (2022) [24] argued that the enhancement of green production technology can promote the clean production of enterprises while alleviating the pressure of end-of-pipe governance, enabling the achievement of green transformation.

Although these studies provide rich perspectives, there remains a notable gap in research concerning the autonomous promotion of green transformation by private enterprises. In contrast, SOEs, as pivotal economic entities, are not only focused on achieving economic gains but are also obligated to fulfill social responsibilities, respond proactively to national policies, and implement national development strategies. In light of the above, this study endeavors to leverage state-owned capital as a vantage point to examine the influence of state-owned equity participation on the green transformation of PEs, thereby offering innovative perspectives and policy recommendations for pertinent practices.

## 3. Theoretical analysis and research hypotheses

### 3.1 The impact of state-owned equity participation on the green transformation of PEs

The participation of SOE equity can build a diversified shareholding structure in private enterprises, and given the unique attributes of the SOE equity system, it can provide a degree of support to private companies through financial, policy, and innovative incentives, thereby exerting a positive influence on the green transformation of PEs. In theory, the influence of SOE equity participation in the green transformation of PEs is mainly reflected in two ways: the resource effect and the governance effect.

(1) Resource effect analysis. The involvement of SOE equity brings more government subsidies, reputational security, and green innovation resources to private enterprises. Firstly, state-owned enterprises, being under state control and managed or overseen by government agencies, are better able to comprehend and leverage relevant policies and support incentives in various sectors, which will help private enterprises secure market resources, tax incentives, and government subsidies, thereby alleviating financial pressures and boosting their green transformation initiatives (Li et al., 2021) [6]. Secondly, state-owned capital participation can convey positive signals to private enterprises, provide reputational guarantees, and enhance investor confidence, thus attracting additional investment bodies, filling the cash flow deficiencies of the green transformation process, and strengthening the capacity of private enterprises to pursue sustainable practices (Abdeljawad et al., 2024) [7]. Thirdly, given the substantial resources and strength of SOEs, their involvement can introduce advanced green technologies and human resources to private firms, expand their relational networks, catalyze collaborative innovation, diminish R&D costs and risks, and expedite the green transformation process of private enterprises (Li et al., 2023) [8].
(2) Governance effects analysis. When SOE equity participates in private enterprises, they can establish an effective system of checks and balances with the majority shareholders of private enterprises, thus exerting positive governance effects. Current research on equity checks and balances within the context of mixed ownership reform generally yields favorable assessments based on a linear relationship. Firstly, equity checks and balances have suppressed the situation of “one-share dominance” in PEs and reduced the shortsighted behavior of management in pursuit of short-term interests. Secondly, SOEs shoulder the historical mission of achieving the strategic goal of national green development, and their shared participation also motivates private enterprises to actively undertake social responsibility, prioritize environmental protection and green development, and facilitate private enterprises’ transition to green practices (Yu & Qi, 2022) [11]. Thirdly, SOEs represent the national image, and private enterprises involved in mixed reform will inevitably be subject to extensive attention and supervision by the media and the public. With the increasing public awareness of environmental protection, the expectations for corporate green development also escalate, compelling enterprises to accelerate the process of green transformation (Chen et al., 2022) [19].

The resource effect and governance effect of SOE equity participation synergistically promote the green transformation of PEs. The resource effect lays the material foundation for green transformation by providing policy support, capital investment, and innovative resources. Meanwhile, the governance effect ensures that these resources are effectively utilized towards green development objectives through equity checks and balances, promotion of social responsibility, and public monitoring. The two are complementary: on the one hand, sufficient resources provide the implementation conditions for effective governance; on the other hand, robust governance ensures that resources are allocated and used properly. Together, they form a dynamic mechanism to promote the green transformation of PEs. Consequently, the involvement of SOE shareholders not only directly enhances the green innovation capability of PEs, but also improves their commitment to and implementation of green transformation by optimizing the governance structure, thus accelerating the greening process of PEs as a whole.

Combining the above analyses, hypothesis H1 is proposed

H1: The SOE equity participation can effectively facilitate the green transformation of PEs.

### 3.2 Analysis of intermediation mechanisms for financing efficiency

According to the signaling theory, private enterprises often face higher financing costs due to greater operational uncertainty, while the participation of SOEs can send positive signals to the market, which not only indicates that the enterprise has good development prospects and lower risks but also effectively alleviates the problem of information asymmetry. At the same time, state-owned capital participation increases the degree of linkage between the interests of the government and enterprises, provides policy support for private enterprises, and improves the social reputation and credit rating of private enterprises, which makes enterprises face fewer financial constraints in obtaining financing (Wang et al., 2023) [25]. When private enterprises receive state capital participation, their social status and reputation are enhanced, and the credit discrimination situation is improved, which reduces the cost of debt financing, expands the scale of debt financing, and effectively alleviates the “difficult financing” and “expensive financing” dilemmas of PEs, which in turn improves the financing efficiency of PEs.

Financial efficiency is an important indicator of a company’s ability to access and use funds, which directly affects its willingness to invest in green transformation. On the one hand, higher financing efficiency means that companies can access funds at a lower cost, reducing the pressure on management to manage surpluses to meet debt covenants, which in turn provides more resources and space for green transformation (Sun et al., 2024) [15]. On the other hand, since green innovation is the key to increasing enterprise value and improving core competitiveness, the improvement of financing efficiency helps to avoid the shortsighted tendency of management, which makes them pay more attention to the long-term development of the enterprise and tends to prioritize the saved funds into green innovation activities to ensure the sustainable development and market-leading position of enterprises (Bo & Yang, 2020) [20]. Therefore, by improving the financing efficiency of private enterprises, the participation of SOE shareholders broadens the source of funds for green innovation activities of enterprises and motivates the management to allocate more resources to green innovation, thus accelerating the realization of green transformation of PEs.

Combining the above analyses, hypothesis H2 is proposed

H2: SOE capital participation promotes the green transformation of PEs by increasing financing efficiency.

### 3.3 Analysis of intermediary mechanisms for environmental information disclosure

According to legitimacy theory, companies will proactively disclose environmental information to demonstrate their commitment and practice to environmental protection in order to gain recognition and support from external stakeholders. The involvement of SOE shareholders can reinforce this need for legitimacy, as SOEs tend to be subject to stricter environmental regulations and social expectations, and this pressure leads SOEs to be more transparent and proactive in their environmental disclosure to maintain their public image and legitimacy (Krammer, 2017) [26]. When state capital participates in private firms, this concern and scrutiny for environmental responsibility are also transferred to private firms, pushing them to improve the quality of environmental disclosure. In addition, according to stakeholder theory, a good corporate governance structure can effectively supervise management to actively disclose environmental information and protect shareholders’ interests. The participation of SOE shareholders tends to optimize the board structure and improve the independence and monitoring function of the board, thereby promoting higher-quality environmental information disclosure. This improved governance structure helps companies better respond to external stakeholder pressure to integrate environmental strategies into corporate decision-making, thereby improving the quality and transparency of environmental information disclosure.

Environmental information disclosure (EID), as a public participatory environmental regulatory tool, has a positive impact on the green transformation of PEs. On the one hand, EID enables environmental regulators to accurately capture the environmental behavior and pollution emissions of enterprises. This increased transparency enhances companies’ awareness of their environmental responsibilities and encourages them to consciously fulfill their social responsibilities. At the same time, regulatory pressure forces companies to increase their investment in green innovation activities (Yu et al., 2019) [16]. On the other hand, EID provides the government, investors, and other stakeholders with the efforts and results of companies’ environmental emission reduction, enabling companies to obtain emission reduction incentives from the government and enhancing investor confidence, thus providing important financial support for the green transformation of PEs. At the same time, enterprises that actively engage in environmental disclosure are more likely to attract environmentally sensitive customers and are even willing to pay a premium to satisfy their demand for green products, which creates external pressure and encourages enterprises to maintain their competitive advantages by continuously improving production processes and innovating green products, thus providing a strong impetus for the green transformation and sustainable development of PEs (Feng et al., 2024) [21].

Combining the above analyses, hypothesis H3 is proposed:

H3: SOE capital participation promotes the green transformation of PEs by improving the quality of environmental information disclosure.

### 3.4 The moderating effect of management compensation incentives

Under the principal-agent theory, while the division of powers facilitates the effective functioning of organizations, it simultaneously results in insufficient oversight by shareholders regarding the actions of management. Green innovation initiatives pose substantial challenges to management decision-making due to the large initial investment, long payback period, potential technological bottlenecks, and elevated uncertainty. As the actual controllers of business activities, managers are frequently compelled to prioritize short-term performance and returns, inclined to succumb to myopic opportunistic behaviors, overemphasizing immediate gains while neglecting the enduring value of corporate green transformation. However, a company’s development depends not only on its own resources or external environment but also on the wise leadership and active promotion of its managers (Wang & Chen, 2018) [22]. As a pivotal internal governance mechanism, management compensation incentives are playing an increasingly important role. A well-designed compensation structure can not only balance the interests of shareholders and management, thereby mitigating the principal-agent dilemma. Furthermore, it can direct management’s attention toward the long-term growth of the organization, proactively discouraging short-sighted behaviors and decision-making (Romec, 2023) [27]. This incentive mechanism facilitates management’s comprehensive understanding of the enterprise’s long-term development, motivating active engagement in sustainable development practices such as green innovation and corporate social responsibility, thereby generating more lasting and stable value for the company.

Academics have posited that both monetary compensation incentives and equity incentives can effectively enhance the alignment of interests between management and shareholders, thus significantly promoting corporate green innovation activities. Scholars such as Chen and Bao (2024) [28] empirically demonstrated that there exists a notable positively correlated relationship between management compensation incentives and corporate green innovation. When management compensation is reasonably enhanced, it will consequently direct more attention to the long-term development of the enterprise. On the one hand, through SOE shareholders, it will introduce investors with positive influence on the green transformation of PEs, accompanied by the provision of financial and technical support, and at the same time put forward the requirements for the green transformation of the enterprise; on the other hand, the participation of SOE shareholders furnishes enterprises with a greater number of policy recommendations about green transformation, prompts the management and SOE shareholders to reach a consensus on the goals, jointly promotes the implementation of the enterprise’s green innovation strategy, fully leverages governance effect of state-owned shareholders, strengthen the supervision and management of PEs through a rigorous regulatory mechanism, and encourages private enterprises to assume greater social responsibility, facilitating a transition from a focus on economic objectives to sustainable development that considers social and environmental benefits, which enables enterprises to expedite the transition to a green economy and contribute to the development of an environmentally sustainable and resource-efficient society (Ding et al., 2023) [23].

Summarizing the above analyses, hypothesis H4 is proposed:

H4: Management compensation incentives play a positive moderating role within the dynamic between SOE equity participation and the green transformation of PEs.

**Fig 2:**
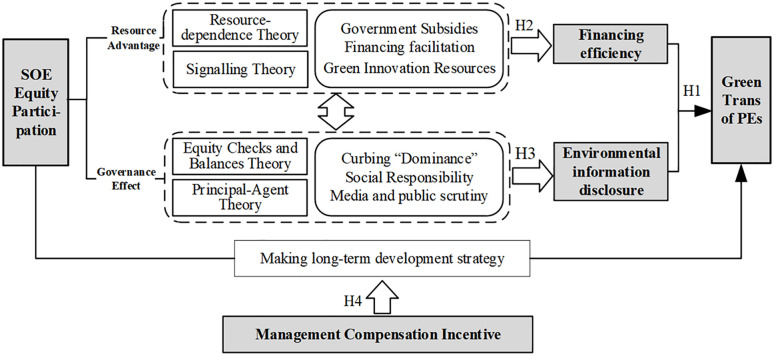
Impact mechanism analysis.

The mechanism of the influence of state-owned equity participation on the green transformation of PEs is shown in Fig 2.

## 4. Research design

### 4.1 Sample selection and data source

The article utilizes data from A-share-listed private enterprises in Shanghai and Shenzhen from 2013 to 2022. To ensure the accuracy of the results, the research object is screened as follows: (1) exclude ST, *ST, PT, and enterprises delisted during the study period; (2) exclude financial institutions; (3) exclude firms experiencing changes in property rights; (4) exclude enterprises with less than three years of listing time; (5) exclude samples with gearing ratio exceeding 1, indicating insolvency. Finally, after screening and organizing the data, 16 139 sample observations are obtained.

The data sources are delineated as follows: (1) Data on corporate green transformation is from the China Research Data Service Platform (CNRDS); (2) Data on the shareholding ratio of the top ten shareholders is from the China Economic and Financial Research Database (CSMAR), and the nature of the shareholders is manually checked and adjusted based on the data of Tianyancha and Qichacha; (3) All other company-level data is from the CSMAR database.

### 4.2 Description of variables

#### 4.2.1 Explained variables.

Regarding the green transformation, literature has been measured through textual analysis, such as Loughran & Mcdonald (2011) [29], which utilized textual information presented in annual reports to evaluate the green transformation of enterprises. Green innovation represents the primary driver of corporate green transformation, so drawing on the research conducted by Yang and Zhao (2023) [30], this study measures green innovation through three types of green patents: the total amount of green innovation, represented by the total amount of independently applied green patents (GrePat), the quality of green innovation, indicated by the independently applied green invention patents (GreInvPat), and the quantity of green innovation, denoted by the independently applied green utility model patents (GreUtyPat) and takes the logarithm of the annual number of green patents applied by the enterprises.

#### 4.2.2 Explanatory variables.

State-owned shareholders’ shareholding. Since public information only provides data on the top ten shareholders, this study focuses exclusively on their proportional representation when constructing explanatory variables. Referring to Yu et al.’s (2022) [11] study on mixed ownership reform, this paper employs a metric defined as the aggregate shareholding ratios of all SOE shareholders among the top ten divided by the total of the shareholding ratios of the top ten shareholders (Soeov). Furthermore, a dummy variable is introduced to indicate whether there is participation from SOE shareholders (Soedum), which serves as an explanatory variable.

#### 4.2.3 Mediating variables.

Financing Efficiency (FE). Following the research conducted by Zhang et al. (2024) [31], this study employs the non-radial super-efficiency DEA model to assess the financing efficiency of enterprises. The input indicators comprise total financing, gearing ratio, and debt-interest ratio, while the output indicators are total asset turnover, net sales margin, and operating income growth rate.

Environmental Information Disclosure (EID). Referring to the study of Liu and Guo (2023) [32], a comprehensive evaluation was conducted on seven key dimensions: environmental management, environmental regulation and certification, environmental performance and governance, environmental liabilities, environmental investment, environmental costs, and environmental information disclosure carriers. In the absence of disclosure, a value of 0 was assigned; for qualitative disclosure only, a value of 1 was assigned; and for qualitative and quantitative disclosure, a value of 2 was assigned. The scores for each indicator for each enterprise were then summed and logarithmically processed to measure the quality of EID.

#### 4.2.4 Moderating variables.

Management compensation incentives (TMTPay). Compensation incentives include monetary and equity incentives, which are designed to motivate management to create more value for the organization. In consideration of the long-term nature of equity incentives, this study employs the natural logarithm of total management compensation as a measure of management compensation incentive intensity.

#### 4.2.5 Control variables.

Referring to the results of existing studies (Zhu et al., 2021; Gao et al., 2024) [33,34], account for several critical variables affecting the green transformation of enterprises: Enterprise size (Size), which measures the overall strength of the enterprise; Return on equity (ROE), which assesses the profitability of the enterprise; Leverage ratio (Lev), which evaluates the liabilities of the enterprise; Dual function (Dual), to measure the efficiency of decision-making and internal control ability; Number of years listed (ListAge), to measure the maturity of the firm; Percentage of fixed assets (FIXED), to measure the efficiency of the firm’s investment and use of fixed assets; Liquidity ratio (Liquid), to measure the firm’s ability to repay short-term debts; and Audit Firm (Big4), to measure the firm’s creditworthiness and financial transparency. Variable definitions in Table 1.

**Table 1:** Variable definitions.

Variable type	Variable	Definition of variables and description of calculations	Unit
Explained variable	GrePat	Ln (count of green invention patent applications + count of green utility model patent applications + 1)	Piece
	GreInvPat	Ln (count of patent applications for green inventions + 1)	Piece
	GreUtyPat	ln (count of green utility model patent applications + 1)	Piece
Explanatory variable	Soeov	The sum of shareholdings of SOEs of the top ten shareholders/ the sum of shareholdings of the top ten shareholders	%
	Soedum	The dummy variable is whether there are state-owned shareholders among the top ten shareholders, taking the value of 1 if there are, and 0 otherwise.	/
Mediating variables	FE	Non-radial super-efficiency DEA model	%
	EID	0 for no disclosure, 1 for qualitative disclosure only, 2 for both qualitative and quantitative disclosure	/
Moderator variable	TMTPay	Natural logarithm of total management remuneration	Yuan
Control variable	Size	The number of employees is taken as a natural logarithm	People
	Lev	Total liabilities/ total assets	%
	ROE	Net profit/ average balance of shareholders’ equity	%
	Dual	The chairman of the board of directors and managing director takes the value of 1, otherwise, it is 0.	/
	ListAge	Ln (current year – year of listing + 1)	Year
	FIXED	Net fixed assets/ total assets	%
	Liquid	Current assets/ current liabilities	%
	Big4	1 if the company is audited by Big 4, 0 otherwise	/
	Year	Annual fixed effects	/
	Ind	Industry fixed effect	/

### 4.3 Model setting

#### 4.3.1 Basic model construction.

In order to evaluate the influence of SOE shareholders on the green transformation of private listed companies (hypothesis H1), constructs a two-way fixed effects model (1) to assess the main regression:


$${Gre}_{it}={\alpha_{\text{0}}+\alpha }_{\text{1}}{Soeov}_{i,t}+\Sigma \alpha_{j}{Controls}_{i,t}+\Sigma Year+\Sigma Ind+\varepsilon_{it}$$ (1)

Where the explanatory variables include GrePat, GreInvPat, and GreUtyPat. The coefficient ‘α_1_’ indicates the total effect of the explanatory variables on the impact of the explained variables. Hypothesis H1 holds if α_1_ is positive and significant. The dummy variable for state-owned shareholders’ participation (Soedum) is also tested in the benchmark regression analysis below.

#### 4.3.2 Mediating effect model construction.

To test the research hypotheses H2 and H3, namely the mediating role of financing efficiency and environmental information disclosure in the green transformation of private enterprises and state-owned equity participation, the following models (2) and (3) are constructed regarding the research of Wen et al. (2014) [35].


$${FE}_{it}={\beta_{\text{0}}+\beta }_{\text{1}}{Soeov}_{i,t}+\Sigma \beta_{j}{Controls}_{i,t}+\Sigma Year+\Sigma Ind+\varepsilon_{it}$$ (2)

$${EID}_{it}={\beta_{\text{0}}+\beta }_{\text{1}}{Soeov}_{i,t}+\Sigma \beta_{j}{Controls}_{i,t}+\Sigma Year+\Sigma Ind+\varepsilon_{it}$$ (3)

#### 4.3.3 Moderating effect model construction.

To evaluate the research hypothesis H4, which pertains to the moderating effects of management compensation incentives on the relationship between state equity participation and green transformation of PEs, Referring to the study by Jiang et al. (2022) [36], the models (4) are constructed as follows. To avoid the collinearity problem after the addition of interactive terms, the variables are centralized.


$${Gre}_{it}={\gamma_{\text{0}}+\gamma }_{\text{1}}{Soeov}_{i,t}+{\gamma_{\text{2}}TMTPay+\gamma }_{\text{3}}{Soeov}_{i,t}*TMTPay+\Sigma \gamma_{j}{Controls}_{i,t}+\Sigma Year+\Sigma Ind+\varepsilon_{it}$$ (4)

## 5. Analysis of empirical results

### 5.1 Descriptive statistics

Table 2 shows the results of descriptive statistics for the primary variables, with a cumulative sample of 16,139 observations. The average value of total green innovation of enterprises (GrePat) is 0.330, reflecting the general green innovation performance of the sampled enterprises; the maximum value extends to 6.050, while the minimum is 0, and the standard deviation of 0.734 suggests a considerable disparity and imbalance in the total green innovation across different enterprises during the sample period. The mean proportion of SOE equity (Soeov) is 0.030, signifying a relatively low incidence of state-owned equity within private firms; the maximum value reaches 0.498, and the minimum is 0. Such a broad range of variation highlights notable variations in the proportion of state-owned shareholding among the sampled enterprises. Indeed, the majority of PEs exhibit a relatively low level of state-owned shareholder participation. Furthermore, the mean value regarding the participation of SOE shareholders (Soedum) is 0.429, which directly reflects that about 42.9% of the private firms in the sample are involved in the mixed ownership reform. The remaining control variables take values within the normal range and are not repeated.

**Table 2:** Descriptive statistics.

Variable	N	Mean	SD	p50	Min	Max
GrePat	16 139	0.330	0.734	0.000	0.000	6.050
GreInvPat	16 139	0.213	0.573	0.000	0.000	5.832
GreUtyPat	16 139	0.199	0.544	0.000	0.000	5.226
Soeov	16 139	0.030	0.063	0.000	0.000	0.498
Soedum	16 139	0.429	0.495	0.000	0.000	1.000
Size	16 139	7.508	1.108	7.428	4.190	11.180
Lev	16 139	0.386	0.188	0.375	0.046	0.908
ROE	16 139	0.060	0.143	0.072	−0.926	0.437
Dual	16 139	0.391	0.488	0.000	0.000	1.000
ListAge	16 139	1.977	0.686	1.946	0.693	3.401
FIXED	16 139	0.188	0.129	0.169	0.002	0.719
Liquid	16 139	2.630	2.393	1.846	0.268	19.370
Big4	16 139	0.035	0.184	0.000	0.000	1.000

### 5.2. Multiple regression analysis

#### 5.2.1 The impact of SOE equity participation on the green transformation of PEs.

Table 3 presents the effects of SOE equity participation on the green transformation of private firms. The findings in columns (1)-(3) illustrate that when the explanatory variable is Soeov, the involvement of SOE shareholders positively affects the GrePat, GreInvPat, and GreUtyPat of private firms, with regression coefficients exceeding 0.282 and exhibiting significance at the 1% level. Columns (4)-(6) show the regression outcomes when the explanatory variable is Soedum, with all coefficients demonstrating significant positive values at the 1% level, indicating that SOE shareholder participation significantly promotes the green transformation of PEs, which confirms hypothesis H1. The reason is that the motivation for “reverse mixed reform” of private enterprises is mainly resource acquisition, industrial synergy, and governance optimization. Specifically, state-owned capital participation serves dual purposes: it aligns with policy demands, prompting SOEs to expedite the green transformation of PEs, and it enables private enterprises to leverage the resource advantages provided by SOEs, which can subsequently be translated into increased green innovation output.

**Table 3:** Analysis of benchmark regression.

Variable	(1)	(2)	(3)	(4)	(5)	(6)
	GrePat	GreInvPat	GreUtyPat	GrePat	GreInvPat	GreUtyPat
Soeov	0.422***	0.333***	0.259***			
	(4.997)	(4.935)	(4.099)			
Soedum				0.068***	0.044***	0.051***
				(5.973)	(4.755)	(5.934)
Size	0.116***	0.091***	0.067***	0.112***	0.089***	0.065***
	(19.454)	(19.175)	(15.102)	(18.755)	(18.618)	(14.409)
Lev	0.293***	0.207***	0.218***	0.299***	0.211***	0.222***
	(6.684)	(5.908)	(6.655)	(6.837)	(6.042)	(6.796)
ROE	0.282***	0.198***	0.196***	0.271***	0.191***	0.188***
	(6.844)	(6.006)	(6.370)	(6.574)	(5.797)	(6.096)
Dual	0.017	0.019**	0.011	0.017	0.019**	0.010
	(1.546)	(2.160)	(1.300)	(1.482)	(2.090)	(1.253)
ListAge	−0.088***	−0.052***	−0.061***	−0.092***	−0.053***	−0.065***
	(−9.576)	(−7.089)	(−8.988)	(−9.920)	(−7.201)	(−9.483)
FIXED	−0.342***	−0.357***	−0.127***	−0.343***	−0.356***	−0.128***
	(−6.645)	(−8.673)	(−3.302)	(−6.665)	(−8.660)	(−3.347)
Liquid	0.003	0.004	0.001	0.002	0.004	0.000
	(0.819)	(1.605)	(0.353)	(0.678)	(1.502)	(0.204)
Big4	0.130***	0.149***	0.040*	0.127***	0.148***	0.037*
	(4.324)	(6.229)	(1.781)	(4.229)	(6.171)	(1.669)
Year	Yes	Yes	Yes	Yes	Yes	Yes
Industry	Yes	Yes	Yes	Yes	Yes	Yes
_cons	−0.461***	−0.426***	−0.272***	−0.445***	−0.418***	−0.257***
	(−9.533)	(−11.017)	(−7.513)	(−9.158)	(−10.754)	(−7.101)
*N*	16139	16139	16139	16139	16139	16139
*R* ^2^	0.167	0.133	0.150	0.169	0.134	0.151
F	92.633	85.799	64.754	95.578	87.676	67.590

Note: 1) ***, **, and * denote significance levels of 1%, 5%, and 10%, respectively; 2) t-statistics are presented in parentheses. Same as below.

#### 5.2.2 Analysis of the mediating mechanism of financing efficiency and environmental information disclosure.

Table 4 presents the regression results of models (2) and (3), which are employed to assess hypotheses H2 and H3, namely, whether financing efficiency and environmental information disclosure serve as mediating factors in the relationship between state-owned equity participation and private enterprises’ green transformation. The results demonstrate that the regression coefficients associated with Soeov are all significantly positive at the 1% level, indicating that state-owned equity participation can facilitate the green transformation of private enterprises by enhancing financing efficiency and the quality of environmental information disclosure. Consequently, Hypotheses H2 and H3 are validated.

**Table 4:** Mediating mechanism test.

Variable	(1)	(2)
	FE	EID
Soeov	0.012***	0.310***
	(3.794)	(4.469)
_cons	1.134***	0.926***
	(654.167)	(24.136)
CVs	Yes	Yes
Year	Yes	Yes
Industry	Yes	Yes
N	16139	16139
R2	0.582	0.397

#### 5.2.3 Moderating effect of management compensation incentives.

Table 5 illustrates the regression results of Model (4), which was employed to assess the impact of state-owned shareholders’ equity participation on the green transformation of PEs when management compensation incentives were introduced as a moderating variable. The results demonstrate that the coefficient of Soeov remains significantly positive and that the coefficients of the Soeov*TMTPay (Interact) are all equal to or greater than 0.200 and are significantly positive at the 1% level. This indicates that management compensation incentives significantly enhance the facilitating effect of SOE equity participation on the green transformation of PEs, which aligns with the expectations outlined in the preceding section and verifies Hypothesis H4.

**Table 5:** Moderating effects test.

Variable	(1)	(2)	(3)
	GreInvPat	GreInvPat	GreUtyPat
Soeov	0.355***	0.281***	0.212***
	(4.254)	(4.227)	(3.398)
TMTPay	0.102***	0.079***	0.067***
	(10.151)	(9.778)	(8.901)
Interact	0.412***	0.404***	0.200***
	(4.075)	(5.011)	(2.649)
_cons	−1.879***	−1.521***	−1.199***
	(−13.289)	(−13.486)	(−11.336)
CVs	Yes	Yes	Yes
Year	Yes	Yes	Yes
Industry	Yes	Yes	Yes
N	16139	16139	16139
R2	0.172	0.138	0.154

### 5.3 Robustness test

To affirm the robustness of the empirical findings, this study performs tests by replacing the measures of the explained variable, substituting sample intervals, provincial-level fixed effects, propensity score matching, and instrumental variable method.

#### 5.3.1 Replacing explained variables.

Table 6 delineates the regression outcomes associated with the modification of the explained variable measure. In the baseline regression, this paper employs the proxy of green innovation to gauge firms’ green transformation. In the robustness test, drawing on the research conducted by Ma and Li (2024) [37], this study incorporates dual dimensions of green technological innovation and productivity optimization to measure green transformation. Green innovation is redefined by the count of acquired green patents in place of application counts, encompassing the total count of independently obtained green patents (GreIUig), the number of independently obtained green invention patents (GreInvig), and the number of independently obtained green utility model patents (GreUmig); and the productivity optimization is quantified through the enterprise’s total factor productivity, which is specifically ascertained by the LP and the OP method. The regression analyses presented in (1)-(3) demonstrate a significant positive correlation between SOE equity participation (Soeov) and green technological innovation. Furthermore, Columns (4)-(5) report the regression outcomes of production efficiency optimization, and both calculation methods indicate the impact of SOE shareholders’ involvement on the optimization of production efficiency within PEs. The above findings affirm that the conclusions remain robust after the modification of the explained variable metrics.

**Table 6:** Robustness test of replacing the explained variables.

Variable	(1)	(2)	(3)	(4)	(5)
	GreIUig	GreInvig	GreUmig	TFP_OP	TFP_LP
Soeov	0.369***	0.235***	0.270***	0.304***	0.259***
	(5.196)	(5.253)	(4.342)	(4.181)	(3.652)
_cons	−0.428***	−0.312***	−0.315***	4.850***	4.463***
	(−10.323)	(−11.937)	(−8.660)	(120.403)	(113.881)
CVs	Yes	Yes	Yes	Yes	Yes
Year	Yes	Yes	Yes	Yes	Yes
Industry	Yes	Yes	Yes	Yes	Yes
N	16139	16139	16139	16139	16139
R2	0.166	0.090	0.151	0.531	0.681

#### 5.3.2 Substitution of the sample interval.

The green transformation of private enterprises may be influenced by changes in the macro-environment. The outbreak of the 2019 novel coronavirus exerted a profound impact on China’s economy, concurrently inflicting substantial negative repercussions on the progression of private enterprises. To control the possible interference of the pandemic on the green transformation, this study re-conducts the empirical analyses after excluding the samples in 2020 and 2021 based on the original model. The findings presented in Column (1) of Table 8 indicate the regression coefficient of explanatory variables is 0.540, statistically significant at the 1% level, suggesting that after excluding the samples in 2020 and 2021, the conclusion remains consistent with prior research, namely, the state equity participation can significantly facilitate the green transformation of PEs.

**Table 7:** Results of PSM balance test.

Variables	Unmatched Matched	Mean		Bias(%)	Reduct |bias|(%)	Difference	
		Treated	Control			T-statistics	P-value
Size	U	7.735	7.337	36.2	94.5	22.95	0.000
	M	7.711	7.689	2.0		1.14	0.254
Lev	U	0.396	0.379	9.2	82.9	5.81	0.000
	M	0.396	0.398	−1.6		−0.92	0.358
ROA	U	0.072	0.051	14.9	92.0	9.32	0.000
	M	0.071	0.069	1.2		0.73	0.466
Dual	U	0.361	0.413	−10.7	98.3	−6.72	0.000
	M	0.362	0.362	0.2		0.11	0.915
ListAge	U	2.156	1.843	46.9	95.4	29.42	0.000
	M	2.147	2.162	−2.1		−1.28	0.202
FIXED	U	0.196	0.183	10.5	97.8	6.64	0.000
	M	0.195	0.196	−0.2		−0.13	0.895
Liquid	U	2.534	2.701	−7.0	86.2	−4.39	0.000
	M	2.539	2.516	1.0		0.57	0.568
Big4	U	0.048	0.025	11.9	83.0	7.65	0.000
	M	0.043	0.039	2.0		1.12	0.262

**Table 8:** Other robustness tests.

Variable	(1)	(2)	(3)	(4)	(5)
	Change Interval	Province FE	PSM	First stage	Second stage
	GrePat	GrePat	GrePat	Soeov	EnvrPat
Soeov	0.540***	0.478***	0.304***		0.912**
	(5.973)	(5.826)	(3.780)		(2.000)
Mean_Soeov				−1.192***	
				(−24.750)	
_cons	−0.515***	−0.513***	−0.464***	0.066***	−0.634***
	(−9.749)	(−10.659)	(−9.052)	(6.550)	(−5.791)
CVs	Yes	Yes	Yes	Yes	Yes
Year	Yes	Yes	Yes	Yes	Yes
Industry	Yes	Yes	Yes	Yes	Yes
Province	No	Yes	No	No	No
Cragg-Donald Wald F				612.763[16.38]	
N	12 061	16 139	16 047	16 054	16 054
R2	0.163	0.176	0.172	0.108	0.164

#### 5.3.3 Increasing regional fixed effects.

The extent of green transformation among private enterprises may also be influenced by the regional level, suggesting that a more advanced regional green transformation enhances the likelihood of private enterprises embracing green transformation. Considering the impact of regional disparities, this paper re-runs the regression after introducing provincial-level fixed effects based on the original model. Column (2) of Table 8 presents the results with the addition of regional fixed effects, revealing a regression coefficient of 0.478, significant at the 1% level, suggests that Soeov can still significantly promote the green transformation of PEs after the addition of provincial-level fixed effects, thereby corroborating the prior conclusion.

#### 5.3.4 Propensity score matching (PSM).

To address the potential endogeneity arising from sample selection bias, whereby the characteristics of PEs may influence the relationship between SOE shareholder participation and the green transformation of PEs, this study employs the propensity score matching method for endogeneity testing. Initially, the private enterprise samples are bifurcated into treatment and control groups based on state-owned equity involvement; Subsequently, utilizing all control variables as covariates, the nearest neighbor matching method with a caliper of 0.001 is applied to align treatment with control samples, and the value of calipers was set to 0.001. The results of the matching are shown in Table 7, which shows that there is no significant difference between the covariates after matching and passes the balance test. The standard deviations (Bias) of the covariates were all less than 10%, and the p-values were all greater than 0.1, i.e., all t-tests failed to reject the original hypothesis that there was no significant systematic difference between the treatment and control groups. Ultimately, the matched sample is re-regressed. The regression results in column (3) of Table 8, after controlling for sample selection bias, indicate that state-owned shareholders’ equity participation can promote the green transformation of PEs, with few substantial changes to the conclusion.

#### 5.3.5. Instrumental variable method.

To mitigate the impact of the endogeneity resulting from reverse causality, that is, the higher degree of green innovation in private enterprises increases the likelihood of attracting state capital participation, this study adopts the two-stage instrumental variable regression method to perform the endogeneity test. Drawing on the study of Rauf and Baolei (2025) [38], the mean value of state capital participation in the same industry and year as the enterprise as the instrumental variables for the test. Due to industry demonstration and policy orientation, private enterprises will consider the state capital participation of other enterprises in the same industry when introducing state capital, so the instrumental variable meets the correlation requirement; however, this indicator only reflects the overall level of state capital participation in the industry and does not directly affect the green transformation process of individual enterprises, so it meets the exogeneity requirement. From the results of the weak IV test in Table 8, the Cragg-Donald Wald F-value is greater than the critical value of Stock-Yogo at the 10% level, passing the weak instrumental variable test. The results in column (4) show that the instrumental variables and explanatory variables with endogeneity are all significantly correlated at the 1% level; the regression results in column (5) show that the regression coefficient of Soeov is 0.912 and is significantly and positively correlated at the 5% level, which is consistent with the previous conclusion.

### 5.4. Heterogeneity test

#### 5.4.1 Group test according to the regional differences.

Due to the substantial disparities in the natural environment, economic development level, policies and regulations, and social-cultural attributes across various regions, the relationship between SOE equity participation and the green transformation of PEs may be contingent upon the geographical location of the enterprises. Referring to the study of Zhang et al. (2025) [39], this paper segments the original sample into eastern, central, and western regions for subgroup regression analysis based on the provincial locations of the sample enterprises. Compared with the central and western regions, the eastern region of China has a higher level of economic development, a more diversified industrial structure, and richer resource endowment. In the eastern region, the higher degree of marketization enables SOE capital participation to better integrate financial and innovation resources, improve resource use efficiency, and promote green transformation. At the same time, environmental regulations are stricter, and policy support is stronger in the eastern region, so SOE participation can further strengthen policy incentives, guide enterprises to meet environmental requirements, and promote environmental innovation. In addition, the governance structure of PEs in the eastern region is relatively sound, and the participation of SOE capital can optimize corporate governance and stimulate the potential for corporate innovation. Fierce market competition and high consumer demand for green products and services also encourage enterprises to more actively accept support from SOE capital to promote green transformation. Based on this, this study expects that the SOE capital participation in PEs located in the eastern region will exert a more pronounced influence on facilitating green transformation. The results in columns (1)-(3) of Table 9 indicate that for private enterprises situated in the eastern region, the coefficient of Soeov is 0.605, and significant at the 1% level, while the regression coefficients of enterprises in the central and western regions are statistically non-significant, aligning with the expectation.

**Table 9:** Heterogeneity analysis.

Variable	Regional groupings			Industry Grouping		Size grouping	
	East	West	Mid	HighTech	Non-High	LEs	SMEs
	(1)	(2)	(3)	(4)	(5)	(6)	(7)
Soeov	0.369***	0.226	0.073	0.529***	0.376***	0.473***	0.047
	(3.355)	(0.922)	(0.445)	(4.982)	(3.389)	(3.466)	(0.436)
_cons	−0.548***	0.002	−0.164	−0.718***	−0.072	−0.459***	−0.080
	(−9.638)	(0.012)	(−1.282)	(−12.026)	(−0.998)	(−4.981)	(−1.126)
CVs	Yes	Yes	Yes	Yes	Yes	Yes	Yes
Year	Yes	Yes	Yes	Yes	Yes	Yes	Yes
Industry	Yes	Yes	Yes	Yes	Yes	Yes	Yes
N	12560	1961	1613	10893	5246	8072	8065
R2	0.177	0.190	0.215	0.136	0.285	0.190	0.147
F	73.234	10.512	12.792	93.555	11.574	40.549	23.308

#### 5.4.2 Group test according to industry differences.

Due to the variability in industry characteristics, high-tech industries are more inclined to prioritize technological innovation in their development concepts, potentially influencing the green transformation trajectories of private enterprises across diverse industries. Drawing upon the research conducted by Ali et al. (2023) [40], the original sample was divided into high-tech and non-high-tech enterprises based on industry classification for segmented regression analyses. High-tech enterprises, as pivotal drivers of China’s economic expansion, garner increasing attention from the state and the government, thereby receiving augmented policy support. However, high-tech enterprises frequently encounter challenges such as elevated R&D expenditures and heightened risks associated with technological innovation endeavors, with state-owned capital participation offering them significant resource benefits and alleviating financial strains. Accordingly, this paper expects that the involvement of SOE capital is particularly influential in advancing the green transformation of high-tech PEs. The findings presented in Table 9 (4)-(5) reveal that the coefficients associated with state-owned shareholder participation are all significantly positive, signifying that indicating that SOE capital can foster the green transformation of PEs, irrespective of whether they are high-tech or not. The Fisher’s combination test found that the p-value is 0.007, which is below 0.01, indicating that the disparity in coefficients across groups is statistically significant, permitting a direct comparison of their magnitudes. Notably, SOE capital participation exerts a more pronounced promotional effect on the green transformation of high-tech enterprises compared to non-high-tech firms.

#### 5.4.3 Group test according to the difference in enterprise size.

There are significant differences between enterprises of different sizes in terms of resource endowment, management capacity, and market influence, which may lead to the green transformation of private enterprises being affected by enterprise size. Therefore, the article divides the sample enterprises into large enterprises (LEs) and small and medium-sized enterprises (SMEs) according to the median after taking the logarithm of the total assets of the enterprises. Large enterprises have richer resources for R&D and innovation, stronger risk-bearing capacity, and are more capable of carrying out green innovation activities due to their well-established management systems, close upstream and downstream relationship networks, and policy guidance. In particular, large enterprises can make more effective use of the financial, technological, and policy support provided by SOE capital to promote green technology R&D and application. At the same time, the involvement of SOE capital can optimize the corporate governance structure, improve management efficiency, and introduce stricter environmental management standards, thus promoting green transformation more vigorously. In addition, large enterprises are more likely to receive policy support and market attention, and the participation of SOE capital further strengthens these advantages, enhances the social recognition and market trust of enterprises, and makes them more competitive in green transformation. Consequently, this study expects that state equity participation in large enterprises has a stronger effect on the green transformation. From the results in columns (6)-(7) of Table 9, the coefficient for the large enterprises’ sample is 0.473 and significant at the 1% level, while the coefficient for the SMEs is not significant, aligning with the expectation.

## 6. Conclusion and recommendation

### 6.1 Conclusions

The article conducts an empirical investigation into the influence of SOE equity participation on the green transformation of PEs in Chinese Shanghai and Shenzhen A-share listed private enterprises from 2013 to 2022. The findings include: (1) State-owned equity participation significantly fosters the process of green transformation of PEs, and the empirical results remain consistent after robustness tests including the change of explained variables’ measurement, alterations in sample interval, the provincial-level fixed effect, instrumental variable, and propensity score matching; (2) The mechanism through which SOE equity participation facilitates green transformation in PEs operates via two primary channels: first, it enhances financing efficiency. SOE equity participation provides credit endorsement for PEs, reduces the risk assessment of financial institutions, and thus reduces financing costs. At the same time, with the resource network and policy support of SOE shareholders, private enterprises can obtain more funds, expand the scale of financing, effectively alleviate the problem of “difficult” and “expensive” financing, and provide sufficient financial security for green transformation. Second, improve the quality of environmental information disclosure. SOE equity participation promotes the optimization of the corporate governance structure of PEs, strengthens the independence of the board of directors and its supervisory function, encourages enterprises to actively disclose environmental information, improves the quality of information disclosure, and enhances social trust. High-quality environmental disclosure not only meets the needs of external stakeholders but also encourages companies to take the initiative in fulfilling their environmental responsibilities and promoting green transformation. (3) Management compensation incentives have a significant positive moderating effect on the relationship between SOE equity participation and the green transformation of PEs. Reasonable compensation incentives encourage management to pay more attention to the long-term development of the enterprise, guide the allocation of resources to green transformation, strengthen the fit between the goals of management and state-owned shareholders, prevent short-sightedness in the process of green transformation, and help private enterprises achieve sustainable development. (4) The heterogeneity analysis indicates that the driving effect varies markedly across different geographical regions, industries, and enterprise sizes. Specifically, compared with the central and western regions, non-high-tech industries, and small to medium-sized enterprises, SOE equity participation exerts a more pronounced driving effect on the green transformation in the eastern regions, high-tech industries, and large-scale enterprises.

### 6.2 Related recommendations

Drawing from the research findings, the following recommendations are proposed: (1) The government should manage differentiated equity participation and focus on regional and industrial synergies. The government should formulate differentiated strategies for SOE equity participation based on enterprise location and industry characteristics. In the eastern region and high-tech industries, the government should guide SOE capital to participate in the “technology synergy” mode to build a green technology R&D platform and strengthen innovation support, while limiting the authority of SOE shareholders to technical compliance checks and not intervening in the market operation of enterprises. In the central and western regions and traditional industries, state-owned capital should participate in the “environmental performance bet” mechanism, and set emission reduction targets linked to revenue, if the enterprise fails to meet the standard, the SOE shareholders can temporarily access environmental protection and technology reform decision-making power, and after meeting the standard through the share repurchase or public transfer of orderly withdrawal. In addition, the establishment of a “dynamic decentralization” mechanism, if the enterprise achieves the green target for many years, the proportion of state-owned voting rights will be reduced accordingly, which not only protects the direction of transformation but also releases the vitality of the enterprise. (2) Enterprises should strengthen governance and disclosure and consolidate core responsibility for green transformation. PEs need to improve their internal governance mechanisms with the participation of SOE shareholders. The board of directors should set up a special committee for green transformation, with directors appointed by SOE shareholders, to oversee environmental information disclosure and environmental investment decisions and to ensure the quality of disclosure and implementation of strategies. The management compensation scheme should integrate green performance metrics, directly associating carbon emission reductions and the acquisition of green patents with incentive structures, to mitigate the propensity for short-term profit maximization. In addition, companies can establish a green technology incubation platform with SOE shareholders to promote the market application of R&D results and build the endogenous momentum of green transformation. (3) Financial institutions should innovate financing support and open up green funding channels. They should leverage the credit-enhancing role of SOE equity participation to develop financing tools suitable for green transformation. For private enterprises engaged in mixed reform, banks can provide tiered green credit lines based on the quality of environmental information disclosure to reduce financing costs; the capital market can establish green equity financing channels and give priority to supporting the issuance of transformation bonds. Insurance institutions can design “green technology insurance” to cover the risk of environmental protection projects and enhance the confidence of social capital participation. Through diversified financial resources, policy support for SOE capital can be transformed into market-based financial support to ease the financial constraints of PEs’ green transformation.

### 6.3 Limitations and prospects

The findings enrich the research on the economic consequences of reverse mixed reform and have some practical value for corporate green transformation. However, the article still suffers from the following shortcomings: (1) Due to the limitation of data collection, the study mainly focuses on Chinese A-share listed PEs in Shanghai and Shenzhen, which may limit the generalizability of the findings; (2) It does not adequately consider the impact of policy changes on the green transformation of PEs; (3) Although it points out the regional and sectoral differences, it does not delve deeper into the specific mechanisms behind them.

Future research can be improved in the following ways: First, by broadening the research sample to include unlisted PEs and PEs in different countries or regions. Second, focus on the impact of policy changes on the green transformation of PEs, combining the implementation time, content, and strength of specific policies, and analyzing in depth the interaction mechanism with the participation of SOE shareholders. Finally, we will further explore the mechanisms behind regional and industry differences, such as how the policy environment, resource endowment, and market maturity in different regions, as well as the technological thresholds and degree of market competition in different industries, affect the path and impact of SOE participation on the green transformation of PEs, to provide more targeted suggestions for policy formulation and enterprise practice.

## Supporting information

S1 File Data. (ZIP)
