# Correction: Research on the resource and governance effects of state-owned equity participation: Based on the analysis of the green transformation process and path of private enterprises

**DOI:** 10.1371/journal.pone.0359539

**Published:** 2026-09-28

**Authors:** Haizhi Ren, Yani Wen

The affiliation for the first author is incorrect. The correct affiliation is not indicated. Haizhi Ren is affiliated with: School of Management, Shenyang Institute of Science and Technology, Shenyang, China.
